# Prediction of Gait Impairment in Toddlers Born Preterm From Near-Term Brain Microstructure Assessed With DTI, Using Exhaustive Feature Selection and Cross-Validation

**DOI:** 10.3389/fnhum.2019.00305

**Published:** 2019-09-18

**Authors:** Katelyn Cahill-Rowley, Kornél Schadl, Rachel Vassar, Kristen W. Yeom, David K. Stevenson, Jessica Rose

**Affiliations:** ^1^Division of Pediatric Orthopaedics, Stanford University School of Medicine, Stanford, CA, United States; ^2^Motion & Gait Analysis Laboratory, Lucile Packard Children’s Hospital, Stanford, CA, United States; ^3^Neonatal Neuroimaging Research Lab, Stanford University School of Medicine, Stanford, CA, United States; ^4^Department of Radiology, Lucile Packard Children’s Hospital, Stanford University School of Medicine, Stanford, CA, United States; ^5^Division of Neonatal and Developmental Medicine, Stanford University School of Medicine, Stanford CA, United States

**Keywords:** MRI, DTI, diffusion tensor imaging, very-low-birth-weight preterm infant, toddler gait, gait impairment, motor development, machine learning

## Abstract

**Aim:**

To predict gait impairment in toddlers born preterm with very-low-birth-weight (VLBW), from near-term white-matter microstructure assessed with diffusion tensor imaging (DTI), using exhaustive feature selection, and cross-validation.

**Methods:**

Near-term MRI and DTI of 48 bilateral and corpus callosum regions were assessed in 66 VLBW preterm infants; at 18–22 months adjusted-age, 52/66 participants completed follow-up gait assessment of velocity, step length, step width, single-limb support and the Toddle Temporal-spatial Deviation Index (TDI). Multiple linear models with exhaustive feature selection and leave-one-out cross-validation were employed in this prospective cohort study: linear and logistic regression identified three brain regions most correlated with gait outcome.

**Results:**

Logistic regression of near-term DTI correctly classified infants high-risk for impaired gait velocity (93% sensitivity, 79% specificity), right and left step length (91% and 93% sensitivity, 85% and 76% specificity), single-limb support (100% and 100% sensitivity, 100% and 100% specificity), step width (85% sensitivity, 80% specificity), and Toddle TDI (85% sensitivity, 75% specificity). Linear regression of near-term brain DTI and toddler gait explained 32%–49% variance in gait temporal-spatial parameters. Traditional MRI methods did not predict gait in toddlers.

**Interpretation:**

Near-term brain microstructure assessed with DTI and statistical learning methods predicted gait impairment, explaining substantial variance in toddler gait. Results indicate that at near term age, analysis of a set of brain regions using statistical learning methods may offer more accurate prediction of outcome at toddler age. Infants high risk for single-limb support impairment were most accurately predicted. As a fundamental element of biped gait, single-limb support may be a sensitive marker of gait impairment, influenced by early neural correlates that are evolutionarily and developmentally conserved. For infants born preterm, early prediction of gait impairment can help guide early, more effective intervention to improve quality of life.

**What This Paper Adds:**

• Accurate prediction of toddler gait from near-term brain microstructure on DTI.

• Use of machine learning analysis of neonatal neuroimaging to predict gait.

• Early prediction of gait impairment to guide early treatment for children born preterm.

## Introduction

At near-term age, the infant brain is rapidly developing (Brody et al., 1987; Dubois et al., 2006; Huang et al., 2006; Oishi et al., 2011; Nossin-Manor et al., 2013; Rose et al., 2014). Brain microstructure abnormalities assessed at this age have been found to correlate to neurodevelopment in preterm children, suggesting potential as early biomarkers for neurodevelopmental impairment (Mukherjee et al., 2002; Arzoumanian et al., 2003; Rose et al., 2007, 2009, 2015; Alvarez et al., 2011; Van Kooij et al., 2011; Woodward et al., 2012; Aeby et al., 2013). Although advances in neonatal medicine have improved outcomes among children born preterm, 40% of very preterm infants develop motor impairments such as cerebral palsy (CP) and developmental coordination disorder, rates that are substantially higher than the general population (Williams et al., 2010; Spittle et al., 2011). Neonatal identification of at-risk children could enable high-impact early intervention during optimal developmental periods of rapid growth and neuronal plasticity.

Diffusion tensor imaging (DTI) is a promising neuroimaging technique that reflects white matter (WM) microstructural injury and can be used to assess early brain development. DTI reveals the amount and direction of water diffusion. In the brain, water diffusion is restricted by neural development, in particular, the presence and isotropy of cellular membranes and myelination. Thus, brain DTI can be used as a metric of brain neurodevelopment and organization (Hüppi et al., 1998; Counsell et al., 2002; Basser and Pierpaoli, 2011). DTI quantifies fractional anisotropy (FA), mean diffusivity (MD), radial diffusivity (RD), and axial diffusivity (AD). FA represents the anisotropy of diffusion, i.e., the extent to which water diffuses in one particular direction (Basser and Pierpaoli, 2011); in WM it is altered by fiber coherence, diameter, density, and myelination. MD is the average amount of water diffusion; AD is the amount of diffusion occurring in the dominant direction or primary axis; and RD is the amount of diffusion occurring perpendicular to the dominant direction. Generally, higher FA and AD indicate more developed microstructure, whereas higher MD and RD indicate less developed microstructure. Brain development alters the dynamics of diffusion, e.g., decreased water content, contraction of extracellular space, myelination, and increased coherence of axonal structures (Kinney et al., 1994; Dubois et al., 2008; Nossin-Manor et al., 2013). The DTI metrics of FA, MD, AD, and RD are affected by these changes and therefore reflect brain development and maturation.

For preterm infants, prognosis based structural brain MRI findings have demonstrated partial success (Miller and Ferriero, 2009; Spittle et al., 2011; Hintz et al., 2015; Anderson et al., 2017). Children at high risk, such as VLBW preterm infants, generally undergo neuroimaging as standard-of-care prior to discharge from the neonatal intensive care unit (NICU). Although currently, DTI is not routinely obtained in NICU clinical brain imaging assessment, it is a promising extension of neuroimaging techniques that may better identify WM microstructural injuries affecting early development (Arzoumanian et al., 2003; Rose et al., 2007, 2009, 2015).

We previously reported on neonatal correlates of toddler gait, analyzing near-term DTI in six subcortical WM regions. We analyzed four bilateral regions and two regions of the corpus callosum (CC) which were selected based on previously reported relevance, using standard statistical techniques in the same cohort of VLBW preterm children (Rose et al., 2015). The current study aims to improve the predictive value of DTI assessment at this early age of brain development. Here we include a broader set of brain regions that may more precisely predict gait impairments and ultimately, may inform neuroprotective treatment to improve outcomes for preterm children.

The current study applies exhaustive feature selection and leave-one-out cross-validation of WM in 99 brain regions, including 48 bilateral regions and three regions of the CC, in order to investigate the use of linear statistical models on DTI metrics for early prognosis of toddler gait impairment. A prior study of this cohort employed similar statistical learning methods to investigate prediction of cognitive and motor neurodevelopment, as measured by the Bayley Scales of Infant Development-Third Edition (Schadl et al., 2018). In this study, we employed a supervised statistical learning approach to determine the predictive value of near-term WM microstructure in VLBW preterm neonates in relation to temporal-spatial gait metrics at 18–22 months adjusted age. We hypothesized that applying a more comprehensive approach using DTI metrics of FA, MD, AD, and RD in a subset of three near-term brain regions, identified using statistical learning approach of exhaustive feature selection and cross-validation, would demonstrate higher predictive value for gait impairment at 18–22 months adjusted age, compared to using standard techniques.

## Materials and Methods

Participants born with VLBW (birth weight ≤1500 g), gestational age at birth ≤32 weeks, and no evidence of genetic disorder or congenital brain abnormalities were recruited. 102 infants treated at Lucile Packard Children’s Hospital (LPCH) NICU from 1/1/10-12/31/11 participated, representing 76% of eligible infants admitted over the 2-year period. All parents of eligible infants were approached prior to scheduled routine MRI and written informed consent was obtained for this IRB-approved prospective cohort study. 66 of 102 neonates had successful DTI scans at near-term age, collected at end of routine MRI scan, prior to discharge from the NICU.

Of the 66 neonates who had both near-term MRI and DTI, 52 completed follow-up gait assessment at 18–22 months of age, adjusted for prematurity (Table 1). Gait was assessed for 2–3 walking trials on an instrumented mat, as described previously (Cahill-Rowley and Rose, 2016a). Walking trials included at least four consecutive footfalls with at least one foot always touching the ground. Temporal-spatial gait metrics included walking velocity, step length, step width, and single-limb support as a percent of the gait cycle (SLS). These temporal-spatial parameters are accurately assessed at toddler age, reflect different aspects of gait function such as symmetry, single limb balance, dynamic postural balance, and overall gait function, and are sensitive to differences in gait pattern and impairment (Cahill-Rowley and Rose, 2016a,b). The Toddle Temporal-spatial Deviation Index (Toddle TDI), an assessment which quantifies deviation of temporal-spatial gait parameters from normal and is sensitive to gross motor function in toddlers (Cahill-Rowley and Rose, 2016b), was calculated. Gait impairment was defined as having a gait outcome score worse than one standard deviation from the mean value of a typically-developing cohort (*n* = 42) at 18–22 months adjusted age, previously published (Cahill-Rowley and Rose, 2016a,b).

**TABLE 1:** Demographic and neonatal characteristics of all neonates and neonates with both DTI and gait assessment.

	**All neonates (*n* = 102)**	**Neonates with DTI and gait assessment (*n* = 52)**
Males, n (%)	42 (41)	20 (38)
Females, n (%)	60 (59)	32 (62)
GA (weeks) mean (SD)	28.7 (2.4)	29.0 (2.4)
BW (g) mean (SD)	1087.3 (278.8)	1081.5 (270.0)
Maternal age (years) mean (SD) (*n* = 99; 51)	31.6 (6.0)	32.1 (6.0)
PMA at scan (weeks) mean (SD) (*n* = 102)	36.6 (1.8)	36.5 (1.2)
Multiple gestation mean (SD)	1.7 (1.0)	1.9 (0.9)
Apgar at 5-minute mean (SD) (*n* = 100; 51)	7.4 (1.9)	7.5 (1.8)
Days on ventilation mean (SD) (*n* = 99; 51)	11.1 (18.4)	8.5 (14.7)
BPD^a^, n (%)	30 (29)	15 (29)
NEC, n (%)	14 (14)	5 (10)
ROP^b^, n (%)	29 (28)	15 (29)
Sepsis^c^, n (%)	12 (12)	5 (10)
Mean CRP^d^, mg/dl, mean (SD) (*n* = 97; 49)	0.45 (0.58)	0.31 (0.35)
Peak CRP^d^, mg/dl, mean (SD) (*n* = 97; 49)	1.00 (1.49)	0.63 (0.84)

*BPD, bronchopulmonary dysplasia; BW, birth weight; GA, gestational age; NEC, necrotizing enterocolitis; PMA, postmenstrual age; ROP, retinopathy of prematurity. ^*a*^BPD: history of respiratory distress syndrome, treated with oxygen >21% at 36 wk PMA. ^*b*^Presence of ROP stage 2 or 3. ^*c*^Sepsis, confirmed by positive blood culture. ^*d*^Measured over first two postnatal weeks.*

### MRI Data Acquisition

Brain MRI scans were performed on a 3T MRI (GE Discovery MR750, 8-Channel HD head coil, Little Chalfont, United Kingdom) at LPCH. A 3-plane localizer was used and an asset calibration was prescribed to utilize parallel imaging. Sagittal T1 FLAIR image parameters were: TE = 9l ms, TR = 2200 ms, FOV = 20 cm, matrix size = 320 × 224, slice thickness 3.0 × 0.5 mm spacing, NEX = 1. T2, DWI, and DTI axial scans were prescribed using a single acquisition full-phase field of view (FOV). The axial fast spin echo T2 imaging parameters were: TE = 85 ms, TR = 2500 ms, FOV = 20 cm, matrix = 384 × 224; slice = 4.0 × 0.0 mm spacing. Axial T2 FLAIR parameters were: TE = 140 ms, TR = 9500 ms, FOV = 20 cm, slice = 4.0 × 0.0 mm, inversion time 2300 ms, fluid-attenuated inversion recovery matrix = 384 × 224. Axial DWI parameters were: TE = 88.8 ms, TR = 10000 ms, FOV = 20 cm, slice = 4.0 × 0.0 mm spacing, matrix = 128 × 128. Coronal T1 SPGR parameters were: TE = 8 ms, TR = 3 s, slice = 1.0 × 0.0 mm spacing, FOV = 24 cm, matrix = 256 × 256.

### Radiological Assessment

Structural MRI was assessed for degree of White Matter Abnormality (WMA) and significant cerebellar abnormality. Radiological evaluation was performed by an experienced pediatric neuroradiologist (XS) and confirmed by a second (KY), both were masked to all other participant data. A form validated for near-term neuroradiological assessment (Hintz et al., 2015) was used to score WMA (1–4) according to a widely used classification system ([Bibr B37][Bibr B15]; Hintz et al., 2015): (i) extent of WM signal abnormality, (ii) periventricular WM volume loss, (iii) cystic abnormalities, (iv) ventricular dilation, and (v) thinning of the CC. High inter-rater agreement (96–98%) for moderate-severe WMA using this classification was reported ([Bibr B37][Bibr B14]). Significant cerebellar abnormalities included significant cerebellar lesions defined by Hintz et al. and/or significant cerebellar asymmetry of ≥3 mm in the anterior-posterior or medial-lateral direction (Hintz et al., 2015). The structural MRI findings in this cohort were previously reported (Rose et al., 2015).

Diffusion tensor imaging was calculated based on diffusion-weighted images (DWI) obtained along 25 orientations with slice thickness of 3 mm, matrix size of 128 × 128, and 90-degree flip angle on a 3T MRI (GE Discovery MR750, 8-Channel HD head coil) at LPCH at the end of routine MRI acquisition. A repetition of DTI sequence was successfully collected in 64 of 66 cases. Thus, in 64 subjects, a full scan was motion free. For 2 out of 66 cases, a composite image was generated by selecting the best slices out of two repetitions manually and combining them to a composite image. Infants were swaddled and fed and typically remained asleep during the scan. Sedation typically was not utilized for routine near-term MRI and was not utilized as part of the research protocol.

### DTI Processing

A trained inspector selected the best DTI repetition to eliminate MRI scans with artifacts or evidence of motion. As noted above, for 2 out of the 66 cases, due to the lack of usable full repetition, a composite repetition was generated from the best image slices. Eddy current distortions were corrected by applying affine transformation. Skull stripping was performed based on B0 and trace (vectorial sum of diffusivity) maps using a ROI editor, and manually rotated to align with the JHU neonatal template, which is a template based on a neonatal brain atlas integrating DTI data with co-registered anatomical MRI (Oishi et al., 2011). Scans were analyzed in a semi-automated, atlas-based manner, using DTI-studio with settings detailed in Oishi et al. (2011).

Diffusion tensor imaging images were processed using DiffeoMap using FA and trace map to perform a large deformation diffeomorphic metric mapping transformation. Amplitude of trace >0.006 mm^2^/s and FA < 0.15 were considered cerebrospinal fluid (CSF) and gray matter, respectively, and were used to obtain the mask of WM regions. WM regions were then segmented into 126 regions based on the JHU parcellation atlas, and the average FA, MD, AD, and RD values were calculated for each region. The number of regions was then narrowed to apical regions ensuring quality of registration, resulting in 48 regions of both sides in addition to the splenium and genu of the CC, and the overall CC (Table 2). Further examination was performed on the FA, MD, AD, and RD values of a total of 99 regions adjusted for postmenstrual age (PMA) at scan.

**TABLE 2:** Brain regions, based on JHU parcellation atlas and segmented using DiffeoMap; DTI metrics (FA, MD, AD, RD) were used as features to find sets of three metrics best predicting outcome.

**Corpus Callosum**	Body of corpus callosum	
	Genu of the corpus callosum	
	Splenium of the corpus callosum	
**Bilateral regions:**	Amygdala	Middle occipital gyrus
	Angular gyrus	Middle temporal gyrus
	Anterior corona radiata	Parahippocampal gyrus
	Anterior limb of the internal capsule	Postcentral gyrus
	Caudate nucleus	Posterior corona radiata
	Cingular part of cingulum	Posterior limb of the internal capsule
	Cingulum gyrus	Posterior thalamic radiation
	Cuneus	Precentral gyrus
	External capsule	Precuneus
	Fornix	Putamen
	Frontal medial orbital gyrus	Retrolenticular capsule
	Fusiform gyrus	Sagittal stratum
	Globus pallidus	Stria terminalis
	Gyrus rectus	Superior corona radiata
	Hippocampal part of the cingulum	Superior frontal gyrus
	Hippocampus	Superior longitudinal fasciculus
	Inferior frontal gyrus	Superior occipital gyrus
	Inferior fronto-occipital fasciculus	Superior occipitofrontal fasciculus
	Inferior occipital gyrus	Superior parietal gyrus
	Inferior temporal gyrus	Superior temporal gyrus
	Insular cortext	Supramarginal gyrus
	Lateral orbitofrontal gyrus	Tapetum
	Lingual gyrus	Thalamus
	Middle frontal gyrus	Uncinate fasciculus

### Statistical Analysis

For each temporal-spatial gait metrics, including velocity, step length, step width, SLS, and Toddle TDI, distinct linear models were generated to examine their correlations with DTI measures. Using an exhaustive search in the feature space, multiple linear regression models were evaluated with leave-one-out cross-validation, L2 regularization, and regularization strength 1.0 to find a set of 3 regions (features) most correlated with gait metrics. Logistic regression models were evaluated with leave-one-out cross-validation, L2 regularization and regularization strength 1.0 on DTI to find a set of 3 regions that best classified high-risk infants scoring worse than one standard deviation from typically developing mean values previously reported (Cahill-Rowley and Rose, 2016a). Best models were selected based on leave-one-out cross-validated, adjusted coefficient of determination (*R*^2^) for linear regression, and leave-one-out cross-validated area under the curve (AUC) of the receiver operator characteristic (ROC) for the binary classification with logistic regression. Logistic regression models were also evaluated on the structural MRI for presence of WMA, cerebellar signal abnormality, cerebellar asymmetry, and intraventricular hemorrhage (IVH, grade 3 or 4) on structural MRI.

Diffusion tensor imaging scalars were adjusted for PMA at scan and normalized to have zero mean and unit variance. To ensure model generalization and robustness, and avoid overfitting, performance measures, i.e., adjusted *R*^2^ and AUC, were evaluated with leave-one-out cross-validation (LOOCV), such that for both regression and classification tasks, N distinct models (*N* = number of subjects) using the same set of features were evaluated leaving out the n-th subject during the determination of the model parameters. Ultimately, each model was evaluated on the left-out subject, and the *N* distinct results in pair with their ground truth values were used to calculate the cross-validated performance metrics. For the classification tasks (high-risk vs. low-risk), balanced sensitivity and specificity were determined by maximizing the sum of the squares of sensitivity and specificity (sensitivity^2^ + specificity^2^). Coefficients of logistic regression reported in Table 3 determine the magnitude and direction with which a feature contributed to the probability of considering a subject as high risk. A positive coefficient implies, that a higher feature value increases the risk, whereas a negative coefficient indicates that a higher feature value lowers the risk, and shifts the evaluation of the logistic function toward the normally developing range. Results were obtained using Scikit-learn (Pedregosa et al., 2011) and Statsmodels (Seabold and Perktold, 2010).

**TABLE 3:** Classification of gait impairment determined by logistic regression of near-term white matter DTI with exhaustive feature selection and leave-one-out cross-validation.

			**Without cross-validation**			**With cross-validation**		
**Variable**	**Brain region (DTI measure)**	**Coefficients**	**AUC**	**Sensitivity**	**Specificity**	**AUC**	**Sensitivity**	**Specificity**
Velocity	R inferior frontal gyrus (FA)	–3.13	0.93	0.93	0.87	0.90	0.93	0.79
	L hippocampus (AD)	1.98						
	Genu (MD)	2.81						
Step width	L lingual gyrus (FA)	–3.13	0.93	0.85	0.85	0.90	0.85	0.80
	L hippocampus (FA)	1.98						
	R putamen (RD)	2.81						
Step length (R)	Genu (MD)	2.22	0.93	0.91	0.88	0.89	0.91	0.85
	L hippocampus (AD)	–3.60						
	R inferior frontal gyrus (FA)	1.64						
Step length (L)	L supramarginal gyrus (MD)	–1.36	0.85	0.93	0.79	0.80	0.93	0.76
	R superior parietal gyrus (AD)	1.32						
	R hippocampus (MD)	–1.29						
Single-limb support (R)	R fusiform gyrus (FA)	–30.68	1	1	1	1	1	1
	Splenium (AD)	61.98						
	Genu (FA)	–43.26						
Single-limb support (L)	R middle frontal gyrus (RD)	–18.83	1	1	1	1	1	1
	R superior occipital gyrus (RD)	47.97						
	L lateral fronto-orbital gyrus (FA)	33.94						
Toddle TDI	R parahippocampal gyrus (RD)	–0.88	0.88	0.90	0.84	0.83	0.85	0.75
	R posterior corona radiata (MD)	–2.21						
	L sagittal striatum (AD)	1.71						

*The three most predictive brain regions for each gait metric are listed. Right (R), Left (L). Significance of all AUC values, *p* < 0.0001.*

## Results

Near-term MRI and DTI were collected at 36.6 ± 1.8 weeks postmenstrual age in 66 children born preterm (28.9 ± 2.3 weeks postmenstrual age) with very-low-birth-weight (1090 ± 266 g). Follow-up gait temporal-spatial parameters were collected in 52 children at 20.2 ± 1.0 months adjusted age; all participants had complete neuroimaging and gait data sets. Table 3 reports the prediction of gait impairment classification based on DTI and MRI using logistic regression with exhaustive feature selection and cross-validation.

**FIGURE 1:**
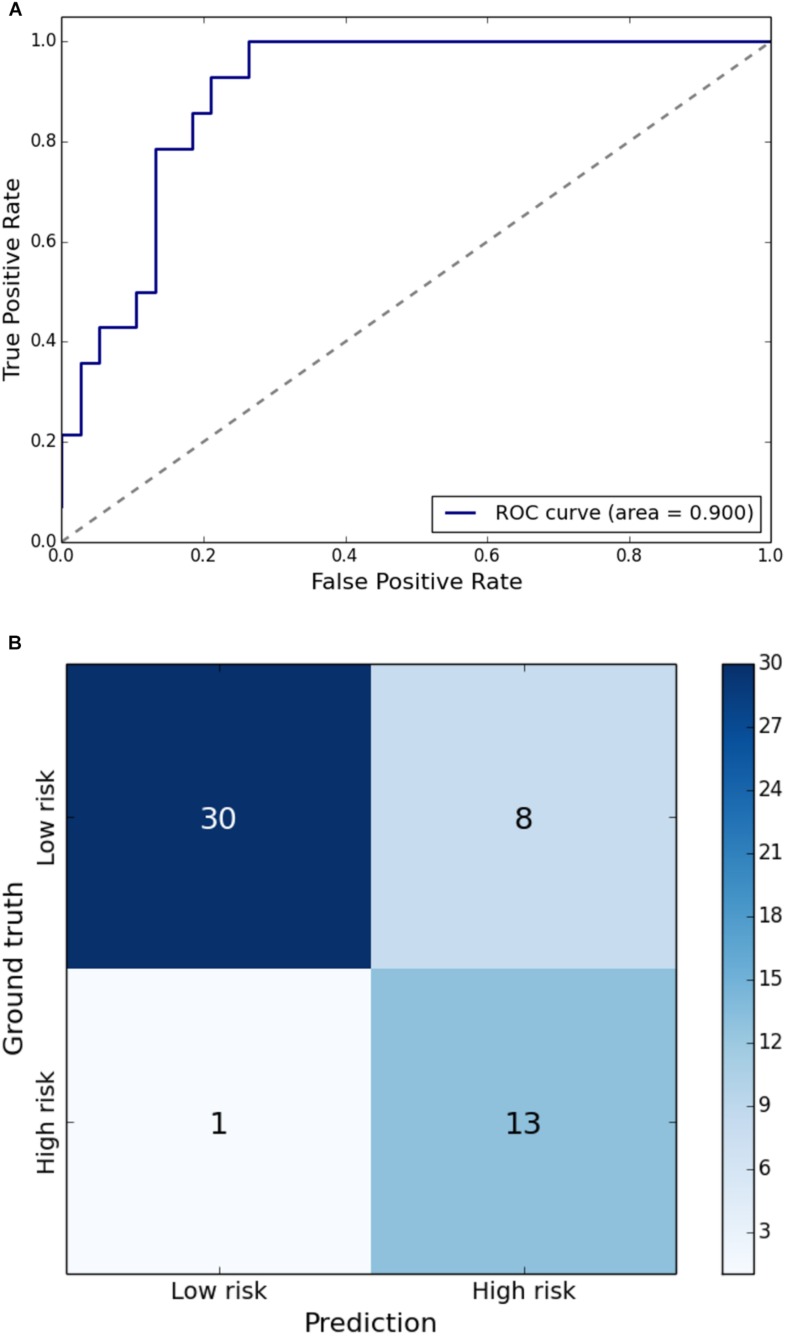
**(A)** Receiver Operating Characteristic curve of leave-one-out cross-validated classification of toddlers having gait velocity below one standard deviation of the mean. **(B)** Balanced confusion matrix of leave-one-out cross-validated classification of toddlers having gait velocity below one standard deviation of the mean.

**FIGURE 2:**
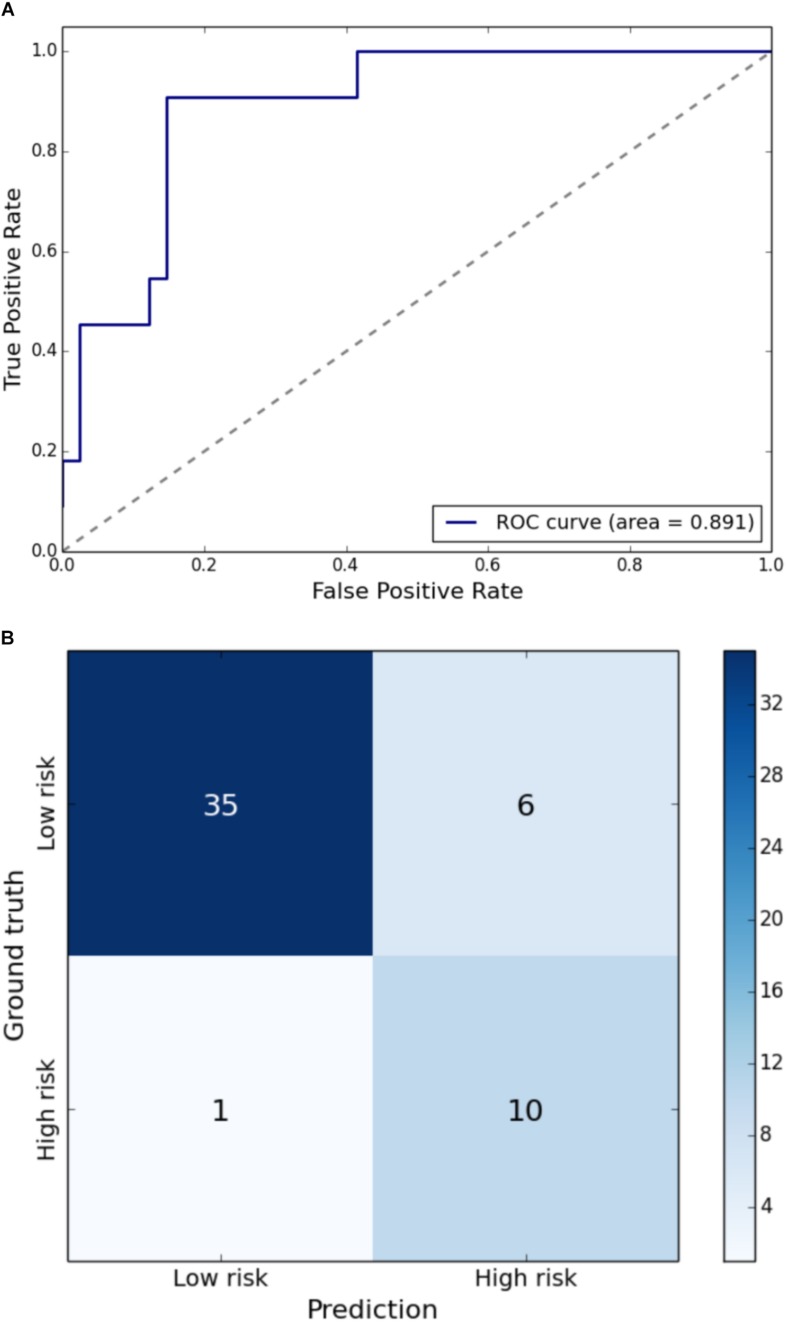
**(A)** Receiver Operating Characteristic curve of leave-one-out cross-validated classification of toddlers having right step length below one standard deviation of the mean. **(B)** Balanced confusion matrix of leave-one-out cross-validated classification of toddlers having right step length below one standard deviation of the mean.

**FIGURE 3:**
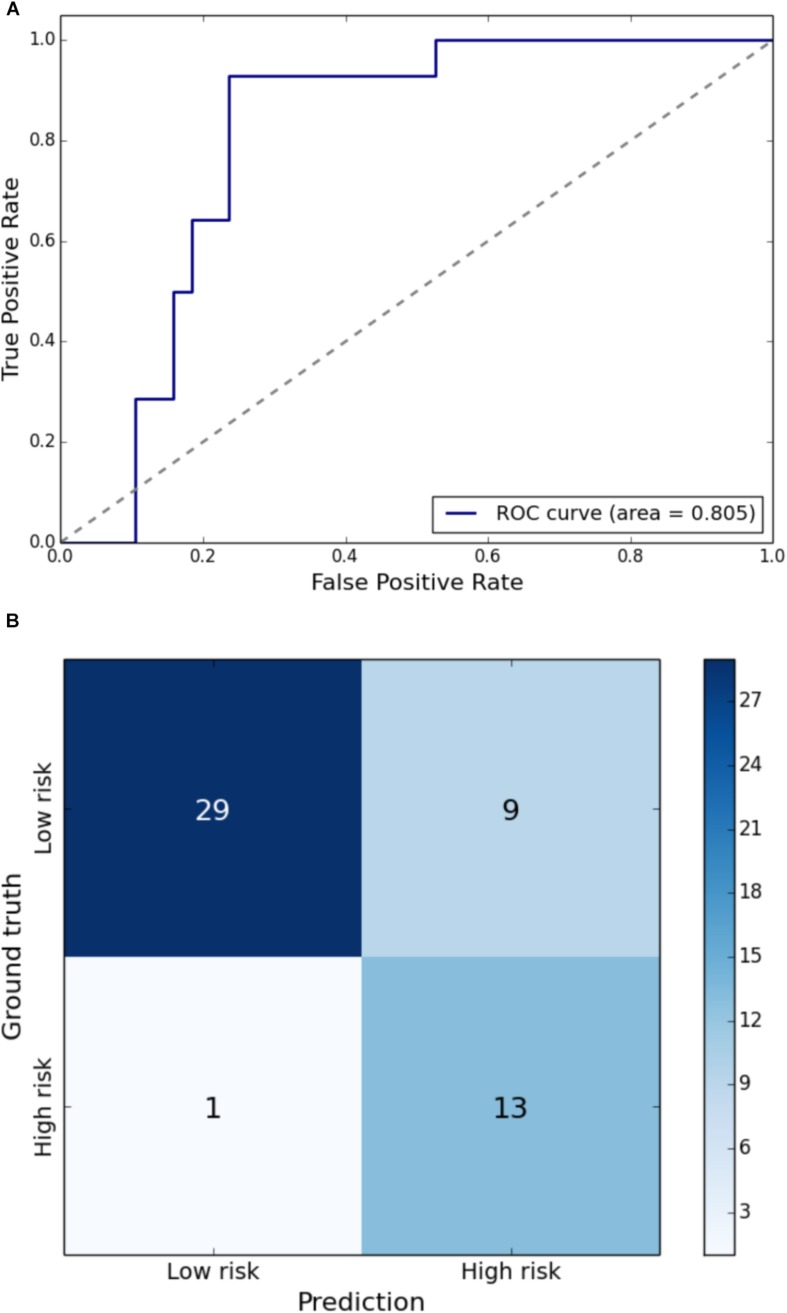
**(A)** Receiver Operating Characteristic curve of leave-one-out cross-validated classification of toddlers having left step length below one standard deviation of the mean. **(B)** Balanced confusion matrix of leave-one-out cross-validated classification of toddlers having left step length below one standard deviation of the mean.

**FIGURE 4:**
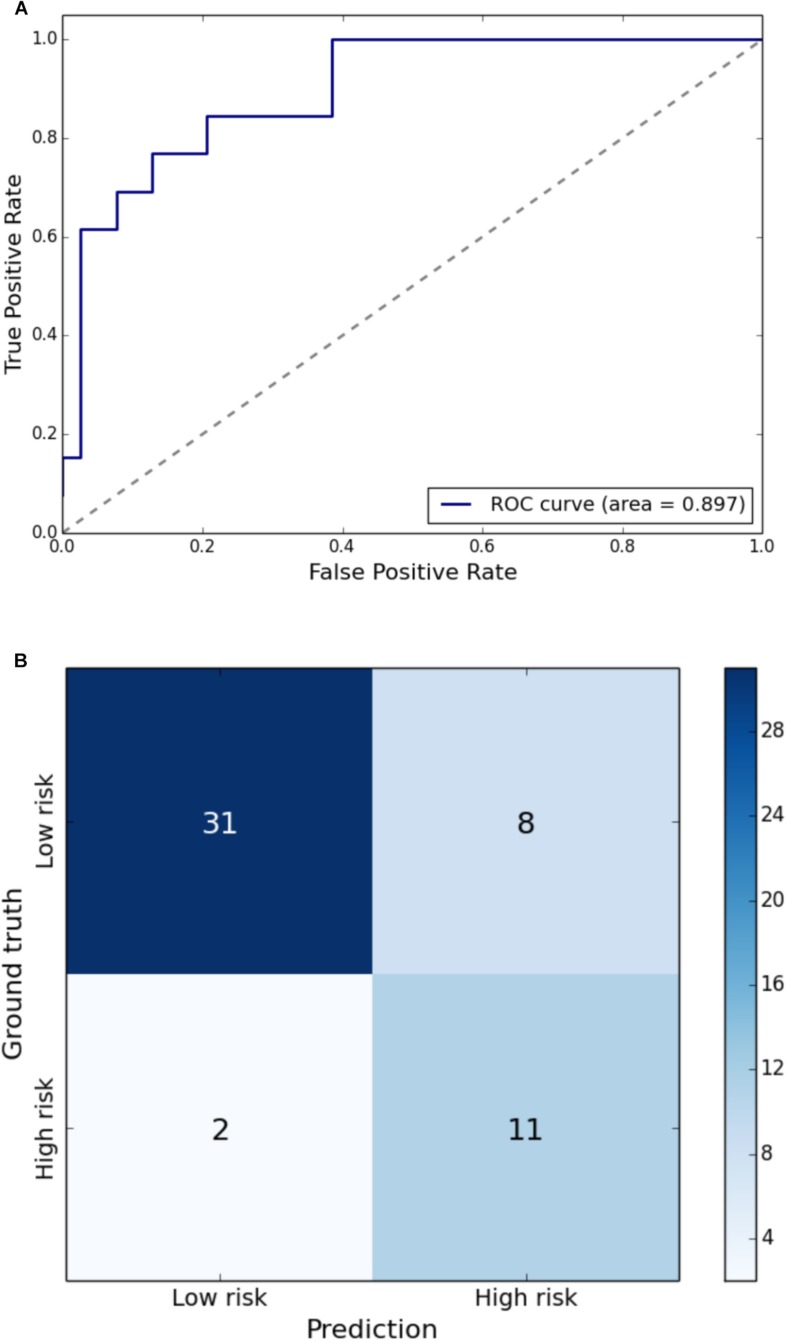
**(A)** Receiver Operating Characteristic curve of leave-one-out cross-validated classification of toddlers having step width above one standard deviation of the mean. **(B)** Balanced confusion matrix of leave-one-out cross-validated classification of toddlers having step width above one standard deviation of the mean.

**FIGURE 5:**
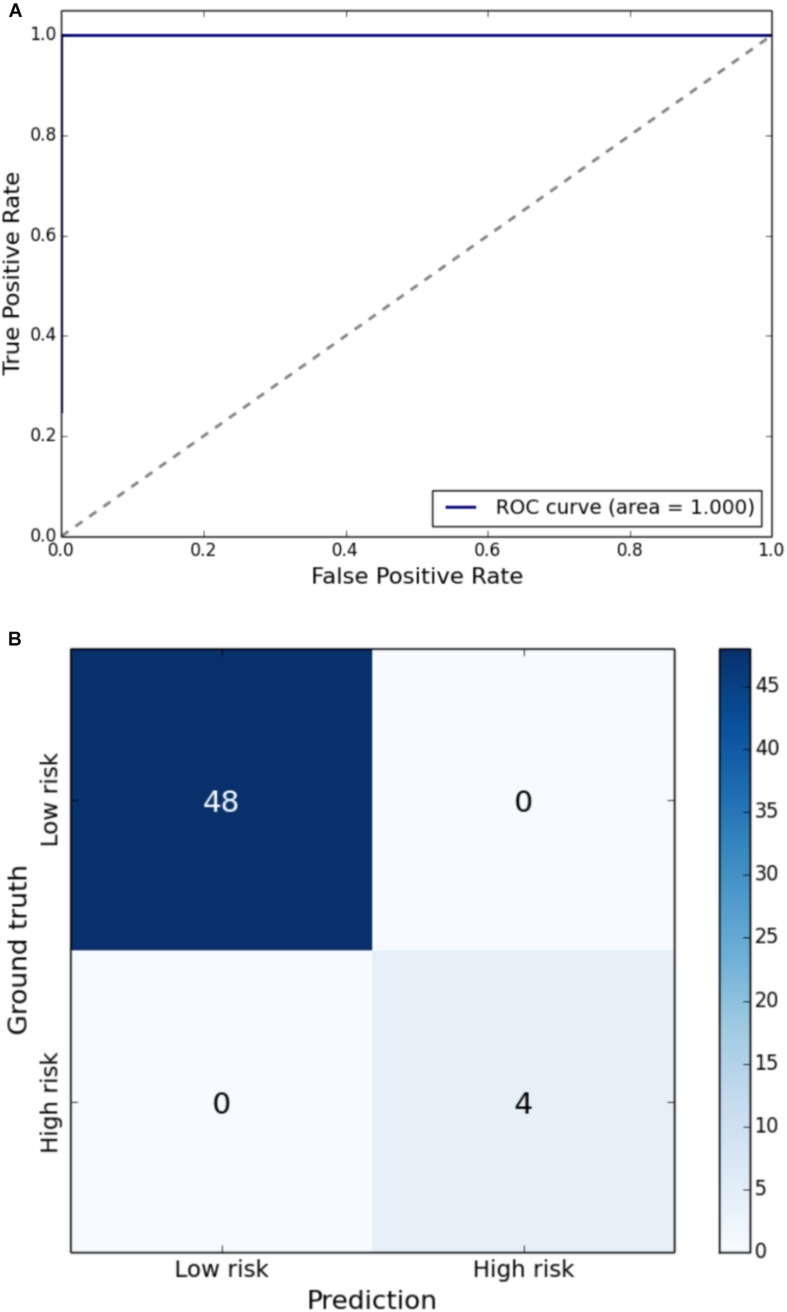
**(A)** Receiver Operating Characteristic curve of leave-one-out cross-validated classification of toddlers having right SLS below one standard deviation of the mean. **(B)** Balanced confusion matrix of leave-one-out cross-validated classification of toddlers having right SLS below one standard deviation of the mean.

**FIGURE 6:**
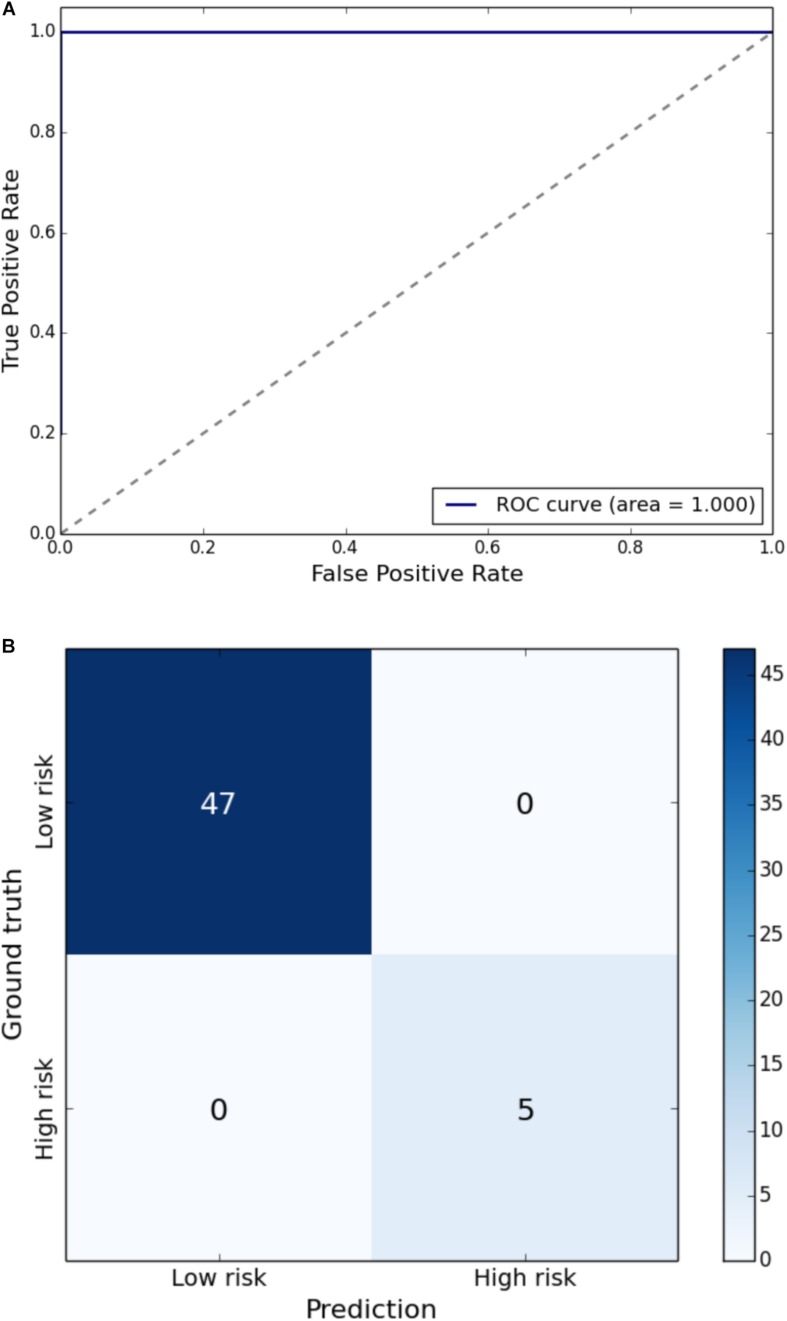
**(A)** Receiver Operating Characteristic curve of leave-one-out cross-validated classification of toddlers having left SLS below one standard deviation of the mean. **(B)** Balanced confusion matrix of leave-one-out cross-validated classification of toddlers having left SLS below one standard deviation of the mean.

Gait impairment was correctly classified for: velocity with 93% sensitivity and 79% specificity (Figures 1A,B); right step length with 91% sensitivity and 85% specificity (Figures 2A,B); left step length with 93% sensitivity and 76% specificity (Figures 3A,B); step width with 85% sensitivity and 80% specificity (Figures 4A,B); right SLS with 100% sensitivity and 100% specificity (Figures 5A,B); left SLS with 100% sensitivity and 100% specificity (Figures 6A,B); and Toddle TDI with 85% sensitivity and 75% specificity (Table 3).

Clinical findings on structural MRI were also evaluated for their predictive value (Table 4) using logistic regression with LOOCV. Presence of WMA, cerebellar signal abnormality, cerebellar asymmetry, and IVH (grade 3 or 4) identified on MRI did not correctly classify children with impaired gait compared to children with typical gait based on TDI; no clinical findings on structural MRI correctly identified more than one child with a particular gait metric abnormality. Logistic regression of near-term structural MRI results did not correctly classify infants as high-risk for impaired velocity, step width, SLS, or step length. Cross-validation revealed that models built on structural MRI assessments, which only included six metrics, were not sufficiently robust to maintain findings with cross-validation. Thus, impairments in gait were not predicted from traditional MRI findings.

**TABLE 4:** Prediction from near-term brain DTI and MRI of gait impairment at 18–22 months.

	**Velocity**		**Step width**		**Step length**				**Single-limb support**				**Toddle TDI**	
					**Right**		**Left**		**Right**		**Left**			
**Methods**	**True +**	**False +**	**True +**	**False +**	**True +**	**False +**	**True +**	**False +**	**True +**	**False +**	**True +**	**False +**	**True +**	**False +**
DTI logistic regression	13/14	5/38	11/13	6/39	10/11	5/41	13/14	8/38	4/4	0/48	5/5	0/47	18/20	5/32
DTI logistic regression with cross-validation	13/14	8/38	11/13	8/39	10/11	6/41	13/14	9/38	4/4	0/48	5/5	0/47	17/20	8/32
White matter abnormality	0/14	3/38	1/13	2/39	0/11	3/41	0/14	3/38	0/4	3/48	0/5	3/47	0/20	3/32
Cerebellar signal abnormality	0/14	6/38	1/13	5/39	0/11	6/41	1/14	5/38	0/4	6/48	0/5	6/47	0/20	6/32
Cerebellar asymmetry	1/14	4/38	1/13	4/39	0/11	5/41	0/14	5/38	0/4	5/48	0/5	5/47	1/20	4/32
Intraventricular hemorrhage, grade 3 or 4	0/14	2/38	0/13	2/39	0/11	2/41	0/14	2/38	0/4	2/48	0/5	2/47	0/20	2/32

*Logistic regression with exhaustive feature selection was able to predict gait impairment, while standard MRI findings were not. Single-limb support (SLS) was perfectly predicted by logistic regression, bilaterally.*

Gait temporal-spatial values were predicted using cross-validated linear regression on near-term DTI with exhaustive feature selection of three brain regions (Table 5). The three most predictive brain regions for gait explained 22% of variance in velocity; 34% in step width; 16 and 15% in right and left step length, respectively; 19 and 16% in right and left SLS, respectively; and 16% of variance in Toddle TDI.

**TABLE 5:** Multiple linear regression models using exhaustive feature selection found the set of three brain regions most correlated with gait metrics.

		**Without cross-validation**	**With cross-validation**
		
**Variable**	**Brain regions**	**Adj. *R*^2^**	**Adj. *R*^2^**
Velocity	L parahippocampal cingulum (RD)	0.32	0.22
	Genu (RD)		
	R inferior frontal gyrus (FA)		
Step width	R tapetum (FA)	0.45	0.34
	L globus pallidus (AD)		
	R globus pallidus (RD)		
Step length (R)	L cingulum cingular part (AD)	0.28	0.16
	L cuneus (FA)		
	L superior occipital gyrus (FA)		
Step length (L)	L supramarginal gyrus (MD)	0.28	0.15
	L inferior temporal gyrus (FA)		
	Genu (RD)		
Single-limb support (R)	L anterior limb of the internal capsule (MD)	0.32	0.19
	Genu (RD)		
	R inferior frontal gyrus (RD)		
Single-limb support (L)	R retrolenticular part of internal capsule (RD)	0.30	0.16
	Genu (RD)		
	R superior occipital gyrus (FA)		
Toddle TDI	L insular cortex (RD)	0.27	0.16
	L inferior temporal gyrus (FA)		
	L anterior limb of the internal capsule (RD)		

*Right (R), Left (L).*

## Discussion

Statistical learning is an area in statistics, referring to a set of tools for modeling complex datasets. It blends parallel developments in computer science, in particular to machine learning, and has been successfully applied to numerous fields. It is also a promising method to improve prognostic accuracy and guide early treatment of preterm infants. We built supervised statistical models using exhaustive feature search applied on near-term brain microstructure assessed on DTI to predict temporal-spatial gait in preterm toddlers at 18–22 months adjusted age. Due to the multiple comparisons inherent to exhaustive search, leave-one-out cross-validation was applied to reduce over-fitting and optimize robustness of generalization. Application of exhaustive feature search with cross-validation on DTI generated relatively high predictive values, compared to standard techniques using structural MRI at near-term age.

Infants were classified with high sensitivity and specificity as high-risk for gait impairment based on near-term WM microstructure (Table 3). Most commonly, the genu of the CC contributed to best performing logistic and linear models (for 3/6 gait parameters and 4/6 gait parameters, respectively) as one of the three selected features, suggesting its strong predictive value for gait metrics. The CC has been previously found to be associated with neurodevelopmental outcome. Anderson et al. (2006) examined 61 VLBW infants and found that poor growth of the CC length was associated with severe motor delay and cerebral palsy by age 2. Mathew et al. (2013) found associations between WM microstructure of the CC as assessed on DTI and motor function in eight very preterm infants. Rose et al. (2008) examined 23 preterm infants and found reduced FA mainly within the posterior regions of the CC. Malavolti et al. (2017) found that adverse motor outcome at 18 months corrected age was associated with smaller neonatal CC size in the posterior subdivision (*p* = 0.003).

Both the hippocampus and the inferior frontal gyrus contributed to several best performing logistic and linear models. The hippocampus contributed to logistic regression of 4/6 gait parameters (Table 3) and is involved in working memory. The inferior frontal gyrus contributed to logistic regression of 3/6 gait parameters (Table 3) and to linear regression of 3/6 gait parameters (Table 5), and has previously been shown to control motor responses (Swick et al., 2008).

Toddler’s with the gait impairment of SLS time were most accurately predicted from near-term brain microstructure. This may be explained because as a toddler learns to walk, achieving sufficient SLS requires single limb strength and balance as well as bilateral stability and symmetry. As a fundamental element of human biped gait, SLS may be a sensitive marker of toddler gait impairment influenced by early neural correlates that are evolutionarily and developmentally conserved. Classification with the logistic function fitted on the right fusiform gyrus FA, splenium AD, and genu FA predicted right SLS with 100% sensitivity and 100% specificity; logistic function fitted on the right middle frontal gyrus RD, right superior occipital RD, and left lateral fronto-orbital gyrus FA predicted left SLS with 100% sensitivity and 100% specificity (Table 3). In addition, linear regression of left anterior limb of the internal capsule MD, genu RD, and right inferior frontal gyrus RD was most predictive of right SLS; and right retrolenticular part of the internal capsule RD, genu RD, and right superior occipital gyrus FA were most predictive of left SLS (Table 5).

Step width was also well predicted in the present study, the linear regression with exhaustive feature search and cross-validation found that the left and right globus pallidus, along with the right tapetum, were predictive of step width, a gait metric that typically reflects development of postural balance.

Coefficients of logistic regression models (Table 3) reinforce prior findings, that in white matter regions with negligible crossing fibers, as compared to gray matter regions, fiber coherence is well measured and reflects neurodevelopment. The direction of the DTI metrics of FA, MD, and AD values of the CC were as expected in affecting the probability of being high risk. Gray matter features provide less ease of interpretation due to higher cortical connectivity, relatively later development, and associated variability in direction of DTI metrics.

We found that prediction by the models using DTI outperformed models using structural MRI (Table 4), consistent with prior studies that found DTI provided higher predictive value for neurodevelopmental outcome compared to structural MRI for Arzoumanian et al. (2003), Rose et al. (2007, 2009), De Bruïne et al. (2013). In our comparison, however, we used metrics that were derived by manual inspection from structural MRI. In further studies, we encourage comparing and examining structural MRI that is segmented and assessed on a regional basis similar to our approach with DTI. Analysis with DTI using the statistical learning approach of exhaustive feature selection and cross-validation has potential to improve prognostic accuracy of neonatal neuroimaging. The clinical feasibility of using DTI is increased by advances in automated data processing that improve its ease of use, repeatability, and thus prognostic value. In this study we used a linear model logistic regression and therefore both its implementation and interpretation are relatively straightforward. These methods could be implemented clinically to improve prognostic accuracy, if replicated in a larger population. Individual patient DTI metrics of most predictive brain regions could be input into a simple spreadsheet to identify infants at high risk for cognitive and motor impairment.

We previously reported data from the same cohort, and evaluated velocity and SLS with respect to WM and cerebellar abnormality as assessed on structural MRI, and in 6 different subcortical WM regions assessed on DTI, using standard partial correlation analyses (Rose et al., 2015). The MRI findings did not correlate with velocity or SLS; genu DTI did correlate with both velocity and SLS. DTI metrics of the other five regions (splenium, anterior limb of the internal capsule, posterior limb of the internal capsule, thalamus, and globus pallidus) did not.

In the current study, the genu and splenium of CC, as well as fusiform gyrus, superior-occipital gyrus, lateral fronto-orbital gyrus, and right middle frontal gyrus contributed to 100% accurate prediction of SLS impairment. Further, the anterior limb of the internal capsule and retrolenticular part of the internal capsule and inferior frontal gyrus also contributed to explaining approximately 15% of the variation of toddler SLS, a sensitive gait metric that reflects gait stability, weight bearing, and symmetry. These findings suggest that a set of brain regions taken together may be more sensitive to outcome than a single region in isolation.

To ensure ease of interpretability we used linear models, which limit accuracy due to the highly non-linear nature of the solution space. Study limitations also include that we analyzed a relatively small cohort which requires the use of statistical tools that are less robust compared to state-of-the-art machine learning approaches (i.e., deep learning), and that classification on imbalanced dataset can be biased, even when using ROC-AUC or precision and recall as performance metrics. Furthermore, evaluation of cortical WM can be confounded by imaging resolution and signal-to-noise ratio. Methods outlined in this study need to be validated on larger preterm populations.

Applying machine learning algorithms on near-term regional WM microstructure may help identify risk of neurodevelopmental delay, guide early intervention and ultimately, may inform neuroprotective treatment to improve quality of life for preterm children. In this study, we employed an exhaustive feature selection algorithm to identify a set of 3 brain regions that best predicted outcomes. Results indicate a relatively high prognostic value for temporal-spatial gait parameters, in particular SLS, and warrant further investigation in larger preterm populations.

## Data Availability

The datasets generated for this study are available on request to the corresponding author.

## Ethics Statement

This study was approved by the Stanford University Institutional Review Board and consent was obtained from parents or guardians.

## Author Contributions

KC-R, RV, JR, and KS contributed to the concept, data collection, data analysis and interpretation, and writing the manuscript. KS, KY, and DS contributed to the concept, data analysis and interpretation, and writing the manuscript.

## Conflict of Interest Statement

The authors declare that the research was conducted in the absence of any commercial or financial relationships that could be construed as a potential conflict of interest.
